# The Effects of Iron Administration on Anemia Development during the 7th and 21st Day of Life in Premature Newborns: A Prospective Cohort Study

**DOI:** 10.3390/medicina60050684

**Published:** 2024-04-23

**Authors:** Oana Cristina Costescu, Aniko Maria Manea, Daniela Mariana Cioboata, Florina Marinela Doandes, Mihaela Zaharie, Mihai Dinu, Daniela Iacob, Marioara Boia

**Affiliations:** 1 Department of Neonatology, “Victor Babes” University of Medicine and Pharmacy Timisoara, Eftimie Murgu Square 2, 300041 Timisoara, Romania; costescu.oana@umft.ro (O.C.C.); cioboata.daniela@umft.ro (D.M.C.); doandes.florina@umft.ro (F.M.D.); mihaela.zaharie@umft.ro (M.Z.); iacob.daniela@umft.ro (D.I.); boia.marioara@umft.ro (M.B.); 2 Doctoral School, “Victor Babes” University of Medicine and Pharmacy Timisoara, Eftimie Murgu Square, No.2, 300041 Timisoara, Romania; 3 Faculty of Medical Engineering, University “Politehnica” of Bucharest, Gheorghe Polizu St., No. 1-7, 011061 Bucharest, Romania; mihai.dinu1411@stud.fim.upb.ro

**Keywords:** iron supplementation, serum ferritin, anemia of prematurity

## Abstract

*Background and Objectives*: The administration of iron to premature newborns is a common intervention aimed at preventing iron deficiency (ID). However, there is no consensus on the optimal timing and dosage for iron supplementation in this population. This study evaluates the effects and potential adverse outcomes of administering iron on the 7th and 21st days of life in premature infants. *Materials and Methods*: This research was conducted on 108 premature neonates at the “Louis Turcanu” Children’s Emergency Clinical Hospital in Timisoara, Romania. The study population was divided into a control group of 48 newborns who did not receive iron supplementation and an intervention group of 60 newborns who did. The analysis utilized univariate and multivariate regression to examine binary outcomes. *Results*: The findings indicate that iron supplementation significantly increased the risk of anemia during the premature period at 21 days of life, as demonstrated by both univariate and multivariate regression analyses, with an odds ratio (OR) of 2.40 (95% CI, 1.01–5.68) and an adjusted odds ratio (AOR) of 2.75 (95% CI, 1.06–7.11), respectively. Contrary to expectations, iron supplementation did not significantly alter the risk of abnormal serum ferritin or iron levels at 21 days of life, according to the univariate analysis (*p* = 0.380 and *p* = 0.526, respectively). *Conclusions*: The observed increase in the risk of anemia without a corresponding improvement in the serum ferritin or iron levels suggests the need for further investigation into alternative strategies for iron supplementation in premature newborns.

## 1. Introduction

Iron is essential for various physiological functions, including organ growth and development across species, with a significant impact on neonatal neurological development. It facilitates myelination, supports neurotransmitter synthesis, and enables the transport of energy metabolites between glial cells and neurons [1]. Studies have demonstrated that iron deficiency impairs myelination and metabolic processes in neurons, particularly within the hippocampus and other brain regions critical for memory processing [2,3]. Additionally, iron deficiency adversely affects the dopamine and opioid systems, along with other brain functions related to memory, learning, thermoregulation, and motor activities [4]. This issue is compounded during fetal and neonatal development, where conditions such as maternal diabetes and placental dysfunction lead to a prioritization of iron for erythropoiesis, thereby reducing its availability for the brain and other organs [5].

Iron deficiency (ID) is the most prevalent nutritional disorder worldwide [4], and congenital ID is a common clinical finding attributed to a variety of risk factors, including maternal diabetes [6,7], obesity [7], alcohol consumption [8], being small for gestational age (SGA) [9], and low birth weight (BW) [10]. The prevalence of ID in preterm newborns stands at approximately 17% [5]. In consequence, iron supplementation should be considered, but its efficacy in newborns for protecting against ID and anemia has been validated in recent systematic reviews [11,12]. However, these reviews also highlight the necessity for further research into the potential long-term effects of iron overload, emphasizing the dual risk associated with both excessive and insufficient iron levels for neurological development [1].

Iron insufficiency impedes hemoglobin biosynthesis, which results in microcytic anemia. The main objectives of iron supplementation are augmenting iron reserves, promoting erythropoiesis, and intensifying systemic oxygen transportation. The transportation of iron across cellular membranes is regulated by the divalent metal transporter 1 (DMT1). Subsequently, iron is stored as ferritin within macrophages and converted into biologically usable Fe^2+^ ions, thereafter being bound to the protein transferrin, which further transports it throughout the organism. Its translocation to the bone marrow is quintessential for erythropoiesis. Moreover, iron is amalgamated with porphyrin and globin chains to produce hemoglobin, which is responsible for the conveyance of oxygen throughout the body [13].

Reports of adverse effects linked to iron supplementation in premature and low-birth-weight newborns are scarce. While most studies have not found significant associations between iron supplementation and neurological development outcomes [14,15,16], there have been observations of increased abnormal neurological examinations in groups receiving late-stage iron supplementation compared to those receiving it earlier [17]. Among the less common adverse effects reported are an increased risk of bloody diarrhea and dysentery [18,19], inhibited growth in length and head circumference [20], decreased weight for age [21], and a higher incidence of respiratory infections [22,23]. Conversely, numerous studies have not shown significant links between iron supplementation and several serious neonatal conditions, including periventricular leukomalacia (PVL) [15,24,25], pulmonary disease [24,25,26], necrotizing enterocolitis (NE) [15,24,25,26,27], and retinopathy of prematurity (ROP) [15,24,25]. Notably, there have been no reports suggesting that iron supplementation increases the risk of anemia during the prematurity period.

Anemia of prematurity (AOP), defined by the development of anemia with an onset that occurs after 6 weeks of life, is characterized as a normocytic, normochromic, hypoproliferative anemia, associated with several postnatal risk factors and conditions, including decreased serum hemoglobin (Hb), hematocrit (HCT), and erythropoietin (EPO) levels, reduced red blood cell (RBC) production and survival, hemorrhage, hypovolemia, and hemolysis [28,29,30].

The variability in iron supplementation strategies, the scarcity of long-term research, and concerns about the adverse effects of iron overload have led to calls for further investigation to determine the optimal dosing and delivery methods [11]. Therefore, this study seeks to evaluate the efficacy and potential side effects of iron supplementation between the 7th and 21st days of life on serum parameters and the incidence of anemia.

## 2. Materials and Methods

### 2.1. Study Design

This prospective cohort study employed a non-randomized clinical trial design conducted from October 2021 to December 2022. The research protocol was approved by the Victor Babeș University of Medicine and Pharmacy of Timișoara Scientific Research Ethics Committee (protocol code number: 79; approval date: 10.11.2021). It comprised two groups: an intervention group, namely newborns who received iron supplementation between the 7th and 21st days of life, and a control group, namely newborns who did not receive iron supplementation. The fact that early iron administration in premature newborns has been shown in multiple studies to reduce anemia and ID incidence [21,24], including two recent systematic reviews [11,12], raised an ethical dilemma in regard to denying an infant treatment when needed if randomly assigned to the control group. Moreover, the “Louis Turcanu” Children’s Hospital treatment protocol advises the administration of iron on an as-needed basis. Due to these ethical considerations commonly associated with this type of research, the study did not utilize randomization. Accordingly, the decision to administer iron was based on a combination of clinical and non-clinical factors, including the infants’ feeding method (breast-fed or formula-fed). Parental or legal guardian consent was secured for all participating newborns, covering the authorization for conducting investigations, performing medical procedures, and the use of data in clinical research. Moreover, the parents or legal guardians were counseled regarding the necessary medical procedures.

This study received ethical approval from the “Louis Tourcanu” Children’s Emergency Clinical Hospital Timisoara Ethics Committee for Scientific Research and Development (protocol code number: 84; approval date: 5.10.2023) and followed the guidelines of the Declaration of Helsinki.

### 2.2. Study Population

This study encompassed premature newborns admitted to the “Louis Turcanu” Children’s Emergency Clinical Hospital in Timisoara, Romania from October 2021 to December 2022. The inclusion criteria included a birth weight (BW) below 2500 g and gestational age (GA) less than 34 weeks. The exclusion criteria included newborns with congenital infections, neurological, cardiac, or renal malformations, genetic syndromes, or hematological conditions such as hemolytic anemia due to microspherocytosis, thalassemia, and group and Rh isoimmunization.

The intervention group comprised newborns who received oral iron supplementation between the 7th and 21st days of life at a dosage of 5 mg/kg BW/day, as per “Louis Turcanu” Children’s Emergency Clinical Hospital treatment guidelines. The decision to administer iron was based on clinical judgment to prevent anemia and iron deficiency, taking into account several perinatal factors, including the feeding method (formula feeding versus breastfeeding). Due to ethical considerations regarding withholding potentially beneficial treatment from premature newborns, randomization was not employed in this study. Instead, newborns were allocated to the intervention group based on a set of predefined clinical criteria aimed at minimizing the risk of iron deficiency while ensuring safety and efficacy. These criteria included indicators of iron stores at birth, the infant’s feeding regimen, and the presence of risk factors for iron deficiency or anemia. Moreover, as per the treatment guidelines, newborns presenting serum EPO levels below 4.3 mUI/mL on the first day of life were administered rhEPO-beta prophylactically and subcutaneously at a dose of 500 U/kg BW at 2, 4, 7, 14, 21, and 28 days of life and, if required, they also received RBC transfusions.

### 2.3. Study Protocol and Definitions

The study sought to investigate the impact of iron on anemia incidence in premature newborns when administered during the first 3 weeks of life. Moreover, the study assessed the relationship between other perinatal factors and incident anemia, such as gestational age (GA), birth weight (BW), APGAR score, and levels of lactate dehydrogenase (LDH), Hb, HCT, RBC, activated partial thromboplastin time (aPTT), prothrombin time (PT), and serum EPO.

The gestational age was determined based on the first day of the last menstrual period and categorized according to World Health Organization (WHO) guidelines into “extremely preterm” (GA < 28 weeks), “very preterm” (28 ≤ GA < 32 weeks), and “moderate to late preterm” (32 ≤ GA < 37 weeks) [31]. The birth weight (BW) was measured immediately after birth and classified following WHO standards into “extremely low birth weight” (<1000 g), “very low birth weight” (<1500 g), and “low birth weight” (<2500 g) [32]. The APGAR scores, assessed at 1 and 5 min post-birth, were categorized as reassuring (7–10), moderately abnormal (4–6), or low (0–3), following the guidelines from the American College of Obstetrics and Gynecology [33].

The hemoglobin, hematocrit, red blood cell count, and serum erythropoietin levels were measured on days 1, 7, and 21 of life. The lactate dehydrogenase level, prothrombin time, and activated partial thromboplastin time were assessed on the first day of life, with reference ranges for PT and aPTT set at 11–14 s and 23–35 s, respectively [34]

Sixty of the one-hundred-and-eight newborns received oral iron supplementation between the 7th and 21st days of life at a dosage of 5 mg/kg BW/day, constituting the intervention group. The decision to administer iron to prevent iron deficiency was based on a range of perinatal factors. Serum iron and ferritin levels were measured at 21 days of life, with normal ranges defined as 10–36 µmol/L for serum iron and 25–200 ng/mL for serum ferritin [35,36]. The classification of anemia and its severity at 21 days was based on Hb levels, following WHO guidelines, where anemia was categorized as mild (Hb 100–109 g/L), moderate (Hb 70–99 g/L), or severe (Hb < 70 g/L) [37].

### 2.4. Statistical Analysis

The statistical analysis in this study was conducted using SPSS Version 23, employing two-tailed tests, with the continuous variables presented as the mean ± standard deviation (SD) and the categorical variables as counts or percentages. Normality tests were performed using the Shapiro–Wilk tests, and the variable distributions were plotted. The Student’s *t*-test and Mann–Whitney U test were used to compare the baseline characteristics represented by the normally and abnormally distributed variables, respectively, while χ^2^ or binomial tests were used to compare the baseline characteristics represented by the categorical variables. Variables such as sex, EPO administration, and iron supplementation were dichotomized for regression analysis, with GA, BW, APGAR scores, aPTT, PT, LDH, Hb, HCT, RBC, and serum EPO levels analyzed as continuous factors in a logistic regression. Anemia was analyzed as both a dichotomized and ranked variable, while the serum ferritin and iron levels were dichotomized. Dichotomization was based on well-known medical definitions and aimed to clarify the effect of the intervention directly on the incidence of the pathology. Univariate and multivariate binomial logistic regression analyses were performed to calculate the odds ratios (ORs) and adjusted odds ratios (AORs) for factors significantly associated with the outcomes. Lastly, *p*-values < 0.05 were deemed significant.

## 3. Results

### 3.1. Comparison of the Baseline Characteristics

The study involved 108 premature newborns with a GA between 24 and 34 weeks and a BW between 650 and 2500 g. Normality tests revealed that with the exception of HCT and RBC, all other continuous variables showed a significant departure from normality (*p* < 0.05). Therefore, to determine if there were significant differences between the intervention and control groups that might impact the results, the baseline characteristics of the newborns were compared using Student’s *t*-test for the HCT and RBC, while the Mann–Whitney U test was performed for the GA, BW, APGAR scores, and day-1 Hb, aPTT, PT, LDH, and serum EPO levels and the one-sample binomial and χ^2^ tests were used to examine the nominal variables, namely sex and congenital anemia. However, as displayed in Table 1, there were no significant differences between the intervention and control groups based on the above baseline characteristics except for the serum EPO levels. The day 1 serum EPO level in the intervention group was 9.30 ± 5.02 mIU/mL, compared to 2.24 ± 1.07 mIU/mL in the control group (*p* < 0.001).

### 3.2. Patient Distribution and Baseline Factors

**Figure 1:**
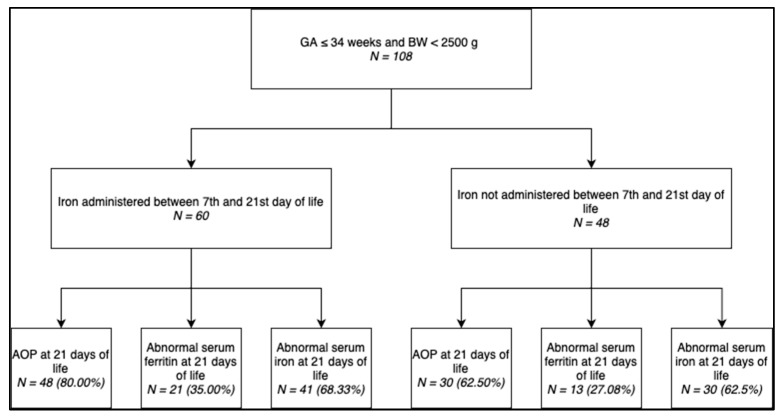
Distribution of newborns according to iron administration status and further stratified by the frequency of anemia and abnormal serum ferritin and iron levels.

Out of the 108 newborns enrolled in this study, 60 received iron supplementation between the 7th and 21st days of life, while 48 were not administered iron. As shown in Figure 1, out of the 60 newborns that underwent iron administration, 48 (80.00%) developed anemia, 21 (35.00%) presented abnormal serum ferritin levels, and 41 (68.33%) presented abnormal serum iron levels, compared to 30 (62.50%), 13 (27.08%), and 30 (62.50%) out of the 48 newborns in the control group, respectively.

Descriptively, the distribution of anemia, abnormal serum ferritin, and abnormal serum iron according to various baseline characteristics, including sex, GA, BW, and APGAR score categories, LDH, aPTT, and PT levels within range, congenital anemia, and EPO and iron administration, can be visualized in Table 2.

The analysis revealed that a high proportion of neonates, both male and female, were diagnosed with anemia by 21 days of life, with prevalence rates of 78.13% in males and 63.64% in females. The incidence of anemia showed a decrease with increasing gestational age (GA) and birth weight (BW). Specifically, 88.89% of the extremely preterm neonates exhibited anemia, compared to 76.36% of the very preterm neonates and 57.14% of the moderate to late preterm neonates. Similarly, the incidence of anemia was 85.71% among the extremely low BW neonates, 74.47% in the very low BW neonates, and 62.50% in the low BW neonates.

A similar decreasing trend in anemia incidence was observed with higher APGAR scores at both 1 and 5 min post-birth. The incidence rates for neonates with APGAR scores of 0–3, 4–6, and 7–10 were 81.82%, 76.47%, and 65.38%, respectively, for the 1 min score, and 100%, 75.56%, and 69.35%, respectively, for the 5 min score. However, this trend did not apply to the incidence of abnormal serum ferritin and iron levels at 21 days of life.

Neonates with normal lactate dehydrogenase (LDH) and prothrombin time (PT) levels on the first day of life showed lower rates of anemia incidence (70.00% and 66.67%, respectively) compared to those with abnormal values (75.00% and 75.76%, respectively). Interestingly, neonates with abnormally activated partial thromboplastin time (aPTT) levels on the first day had a lower incidence of anemia (69.47%) than those within the normal range (92.31%). Additionally, abnormal serum ferritin levels were more common in neonates with normal aPTT levels.

The administration of recombinant human erythropoietin (rhEPO) was associated with lower proportions of anemia, abnormal ferritin, and abnormal iron levels (63.27%, 26.53%, and 61.22%, respectively) compared to those not receiving rhEPO (79.66%, 35.59%, and 69.49%, respectively). Contrarily, neonates who received iron supplementation between the 7th and 21st days of life had higher incidences of anemia, abnormal ferritin, and abnormal iron levels at 21 days (80.00%, 35.00%, and 68.33%, respectively) compared to those not supplemented with iron (62.50%, 27.08%, and 62.50%, respectively).

Following normality tests, the distribution of all independent continuous variables was assessed as normal, with both K–W and Shapiro–Wilk tests showing insignificant differences between a normal distribution and the distribution of the GA, BW, APGAR scores, and day-1 Hb, HCT, RBC, aPTT, PT, LDH, and serum EPO levels.

### 3.3. Univariate Regression Analysis

#### 3.3.1. Anemia Risk

Following the univariate logistic regression analysis, the GA, BW, day-1 Hb and HCT levels, and day-1 RBC count were shown to be protective factors against anemia, while iron administration surprisingly emerged as a risk factor for anemia. As illustrated in Table 3, BW and day-1 HCT levels were associated with small reductions in anemia risk, with respective ORs of 0.999 (95% CI, 0.999–1.000; *p* < 0.05) and 0.930 (95% CI, 0.868–0.997; *p* < 0.05), while the GA and day-1 Hb levels were associated with moderate reductions of approximately 20% in anemia risk, having respective ORs of 0.802 (95% CI, 0.668–0.963; *p* < 0.05) and 0.776 (95% CI, 0.635–0.949; *p* < 0.05), and the RBC count was associated with more than a 3-fold reduction in anemia risk, having an OR of 0.316 (95% CI, 0.139–0.716; *p* < 0.05). Iron supplementation was associated with more than a 2-fold increase in anemia risk, presenting an OR of 2.400 (95% CI, 1.014–5.678; *p* < 0.05).

#### 3.3.2. Abnormal Serum Ferritin and Iron

Following the univariate logistic regression analysis, none of the baseline characteristics or interventions were significantly associated with abnormal serum ferritin levels, while only BW showed a significant association with increased risk of abnormal serum iron levels, yet with a negligible effect size, having an OR of 1.001 (95% CI, 1.000–1.002; *p* < 0.05). As shown in Table 4 and Table 5, all associations with the exception of BW and serum iron were insignificant, having *p*-values > 0.05.

### 3.4. Multivariate Regression Analysis

Given that abnormal serum iron was only associated with BW, multivariate regression analyses were not performed to determine the outcomes of abnormal serum ferritin and iron. However, all factors that were univariately associated with anemia incidence at 21 days in the previous analysis, namely GA, BW, day-1 Hb and HCT levels, day-1 RBC count, and iron supplementation, underwent multivariate logistic regression. As shown in Table 6, iron administration during the 7th and 21st day of life was the only anemia risk factor, being associated with a 2.7-fold increase in anemia risk, corresponding to an AOR of 2.749 (95% CI, 1.064–7.106; *p* < 0.05). The Nagelkerke R square value of 0.226 indicates that 22.6% of the variance in the dependent variable, namely the AOP at 21 days of life, can be explained by movement in this set of predictor variables. The relatively wide confidence interval is specific to small sample sizes, warranting further studies involving larger populations.

## 4. Discussion

### 4.1. Literature Findings

The efficacy of early iron administration in premature newborns, including the reduction of anemia and ID risk and the increase in both serum ferritin and iron levels, has been demonstrated across multiple individual studies [22,25,38,39] and confirmed by the results of meta-analyses in recent systematic reviews [11,12]. However, the main results of this study did not show a significant effect on incident anemia. A recent study found that in study populations that received rhEPO, similar to the neonates in this study, the administration of high doses of iron had no effect on the incidence of ID, while the concomitant administration of rhEPO might have contributed to a significant reduction in serum ferritin levels [40]. Furthermore, it was demonstrated that iron supplementation at 6 weeks of life can cause a decrease in serum ferritin, contrary to the effect of supplementation at 2 weeks of life [15]. In another report, iron administration continued from the 2nd week of life until the end of the 60th day of life and did not increase the serum ferritin levels compared to the control group [24]. Accordingly, a modest performance of iron supplementation in preventing ID has been reported for the past 50 years, and given the lack of consensus regarding optimal dosing, reduced effectiveness may be expected [41,42,43].

Unexpectedly, in our study, iron supplementation showed a significant increase in anemia risk, nearly tripling it with an AOR of 2.749 (95% CI, 1.064–7.106). This direct association has not been previously reported in the literature. However, there have been reports of multiple side effects, most notably bloody diarrhea and dysentery [18,19], which may have had a possible contribution to the increase in anemia at 21 days of life in our study. Although bloody diarrhea and dysentery may have contributed to a reduction in iron supplementation effectiveness, unfortunately, no data were collected in regard to these conditions and, therefore, their impact could not be assessed. Furthermore, iron overload has been recently associated with the increased production of reactive oxygen species (ROS) and lipid peroxidation, leading to ferroptosis and triggering mechanisms that counteract the expected benefits of iron administration [44,45].

No significant differences were detected between the baseline characteristics of the patients in the intervention and control groups, except for day 1 serum EPO, which was markedly increased in the intervention group (*p* < 0.001). There are multiple factors that might have contributed to this difference, including non-significant, slightly increased average BWs and GAs in the intervention group. However, the day 1 serum EPO levels were not significantly associated with anemia at a univariate level, and, therefore, the results were unlikely to have been impacted by this baseline difference.

Several alternative explanations warrant consideration regarding the observed association between iron supplementation from the 7th to the 21st days of life and the incidence of anemia at 21 days of life. Firstly, the broad confidence interval for the adjusted odds ratio (AOR), which exhibits a marginally non-significant lower boundary, suggests limitations due to the small sample size. Increasing the sample size from the same population could potentially shift the lower boundary of the AOR below 1, rendering the association statistically non-significant. Additionally, significant differences in the serum erythropoietin (EPO) levels were noted between the intervention and control groups (9.30 ± 5.02 vs. 2.24 ± 1.07 mIU/mL; *p* < 0.001, respectively). Elevated serum EPO levels in the intervention group may indicate an adaptive physiological response to either hypoxia or anemia, suggesting that iron utilization for red blood cell (RBC) synthesis might be compromised, or an alternate mechanism may be affecting erythropoiesis or increasing hemolysis.

Furthermore, the experimental conditions of this study, including the iron dosage (5 mg/kg body weight/day), the period of administration (7 to 21 days of life), and the timing of outcome measurement (21 days of life), vary substantially from those reported in other studies. The literature indicates a range of enteral iron dosages from 1 mg/kg/day to 4 mg/kg/day [14,24], with supplementation initiation spanning from 7 to 80 days of life [17,23], and outcomes assessed as early as 2 weeks to as late as 7 years [14,25]. This variation highlights the importance of aligning study designs for meaningful comparisons and interpretations across different research works, underscoring the complexity of determining the optimal approach for iron supplementation in the prevention of anemia.

This study reveals surprising and counterintuitive results regarding the benefits of iron administration between the 7th and 21st days of life in preterm newborns. These results warrant further research into multiple aspects of iron administration in preterm newborns. Firstly, it is very important that more studies address the effectiveness of iron in reducing ID risk and contributing to normal serum ferritin and iron levels administered to preterm neonates receiving rhEPO treatment. Secondly, the lack of consensus regarding the optimal dosing and administration schedule of iron in preterm newborns prompts further investigation. Thirdly, it is important to consider multiple-micronutrient (MMN) deficiencies, which can have similarly detrimental effects on ID and require more complex supplementation using MMNs as a possible alternative to iron and zinc supplements [46].

### 4.2. Study Limitations

A significant limitation of the study was the lack of randomization, attributed to ethical concerns that precluded withholding potentially beneficial treatments from premature infants. Consequently, iron was administered based on clinical need, which may introduce bias in evaluating its effects. Additionally, the attrition of participants after the 21st day limited the investigation into the long-term impacts of early iron supplementation on development and iron deficiency status [47]. Moreover, it is possible that 14 days might not be sufficient to accurately detect the impact of iron administration; therefore, an assessment conducted at a later date may have revealed different results. Although a notable baseline difference existed between the groups in terms of day 1 serum erythropoietin (EPO) levels, with higher levels observed in the intervention group, its influence on the study outcomes is considered minimal. This assessment is based on the finding that day 1 serum EPO levels were not significantly linked to anemia in the univariate logistic regression analysis. The study also faced challenges due to its small sample size, which could affect the generalizability of the findings.

It is important to note that the precise functions of ferritin in the intrauterine environment, particularly in regard to development and fetal growth, are not well-defined. Although ferritin concentration in meconium is a promising marker for evaluating fetal homeostasis, further research is required to better understand the function of ferritin in fetal growth. This prompts a more cautious interpretation of the results outlined in this study [48].

Furthermore, the study did not account for whether the infants were fed breast milk or formula, nor did it document the occurrence of diarrhea, factors that could provide additional insights into the results. The data did not include pregnancy-related complications, a possible influencing factor that might have impacted the results. Additionally, the study lacked comprehensive data on critical perinatal characteristics and morbidities, such as maternal iron status and gestational hypertension. The latter has been recently identified as a factor associated with an increased risk of ID, suggesting that these omitted variables could have enriched the understanding of the study’s findings and implications. Moreover, although AOP typically manifests 6–8 weeks post-birth, the role of sideropenia in its pathogenesis may be less direct than initially considered. The choice of anemia as a primary outcome at 21 days, and not specifically AOP, was intended to identify the trends in the hemoglobin and iron levels that could have influenced the longer-term outcomes early.

## 5. Conclusions

This study confirms the limited effectiveness of early iron supplementation in preterm newborns between the 7th and 21st days of life in preventing abnormal serum iron and ferritin levels and presents a novel finding, namely the increased risk of developing anemia at 21 days of life. Therefore, subsequent research on the optimal iron dosing in order to achieve a better benefit/risk ratio is recommended, as well as exploration of alternative nutrient supplementation strategies that can better prevent anemia and ID while presenting fewer risks to newborns.

## Figures and Tables

**Table 1:** Univariate comparison of postnatal characteristics in neonates who were administered iron between the 7th and 21st days of life and those who were not.

Characteristics	Iron administered between the 7th and 21st Days of Life (*n* = 60) *	Iron Not Administered between the 7th and 21st Days of Life (*n* = 48) *	Statistical Test Performed	*p* Value
Male	38 (63.33%)	26 (54.17%)	Binomial	0.169
Female	22 (36.67%)	22 (45.83%)	Binomial	1.000
GA, weeks	30.13 ± 2.30	29.69 ± 2.90	Mann–Whitney	0.474
BW, g	1415.07 ± 401.41	1352.19 ± 466.23	Mann–Whitney	0.371
APGAR score at 1 min	5.53 ± 2.48	5.79 ± 2.06	Mann–Whitney	0.865
APGAR score at 5 min	6.57 ± 1.37	6.54 ± 1.68	Mann–Whitney	0.980
Hb at 1 day of life, g/dL	15.24 ± 2.49	15.51 ± 3.26	Mann–Whitney	0.272
HCT at 1 day of life, %	43.76 ± 6.72	46.00 ± 7.03	Student’s *t*-test	0.095
RBC count at 1 day of life, million/μL	4.13 ± 0.62	4.28 ± 0.64	Student’s *t*-test	0.233
aPTT at 1 day of life, s	52.72 ± 19.45	56.26 ± 38.12	Mann–Whitney	0.658
PT at 1 day of life, s	16.34 ± 4.39	15.80 ± 5.49	Mann–Whitney	0.169
LDH at 1 day of life, U/L	812.45 ± 397.50	715.27 ± 363.91	Mann–Whitney	0.127
Serum EPO at 1 day of life, mIU/mL	9.30 ± 5.02	2.24 ± 1.07	Mann–Whitney	<0.001
Congenital anemia	3 (5.00%)	4 (8.33%)	Binomial	1.000
No congenital anemia	57 (95.00%)	44 (91.67%)	Binomial	0.232

* Categorical variables are represented as *n* (%); continuous variables are displayed as means ± SD; GA, gestational age; BW, body weight; Hb, hemoglobin; HCT, hematocrit; RBC, red blood cell; aPTT, activated partial thromboplastin time; PT, prothrombin time; LDH, lactate dehydrogenase; EPO, erythropoietin.

**Table 2:** Distributions of various clinical conditions grouped by baseline characteristics.

Subgrouping	Total Population (*n*)	Anemia at 21 Days of Life		Abnormal Serum Ferritin Level at 21 Days of Life		Abnormal Serum Iron Level at 21 Days of Life	
		No. of Events	Percentage of Events	No. of Events	Percentage of Events	No. of Events	Percentage of Events
Total	108	78	72.22%	34	31.48%	71	65.74%
Sex							
Male	64	50	78.13%	22	34.38%	42	65.63%
Female	44	28	63.64%	12	27.27%	29	65.91%
Gestational age (wks)							
32–37	35	20	57.14%	12	34.29%	24	68.57%
28–31	55	42	76.36%	18	32.73%	34	61.82%
<28	18	16	88.89%	4	22.22%	13	72.22%
Body weight (g)							
1500–2499	40	25	62.50%	17	42.50%	21	52.50%
1000–1499	47	35	74.47%	13	27.66%	35	74.47%
<1000	21	18	85.71%	4	19.05%	15	71.43%
Apgar 1 min							
7–10	52	34	65.38%	18	34.62%	37	71.15%
4–6	34	26	76.47%	10	29.41%	21	61.76%
0–3	22	18	81.82%	6	27.27%	13	59.09%
Apgar 5 min							
7–10	62	43	69.35%	21	33.87%	46	74.19%
4–6	45	34	75.56%	12	26.67%	24	53.33%
0–3	1	1	100.00%	1	100.00%	1	100.00%
Normal LDH at 1 day of life (135–750 U/L)							
Yes	60	42	70.00%	17	28.33%	39	65.00%
No	48	36	75.00%	17	35.42%	32	66.67%
Normal aPTT at 1 day of life (23–35 s)							
Yes	13	12	92.31%	4	30.77%	11	84.62%
No	95	66	69.47%	30	31.58%	60	63.16%
Normal prothrombin Time 1 day of life (11–14 s)							
Yes	42	28	66.67%	12	28.57%	33	78.57%
No	66	50	75.76%	22	33.33%	38	57.58%
Anemia at 1 day of life							
Yes	7	4	57.14%	2	28.57%	5	71.43%
No	101	74	73.27%	32	31.68%	66	65.35%
EPO administered in first 7 days of life							
Yes	49	31	63.27%	13	26.53%	30	61.22%
No	59	47	79.66%	21	35.59%	41	69.49%
Iron administered within 7–21 days of life							
Yes	60	48	80.00%	21	35.00%	41	68.33%
No	48	30	62.50%	13	27.08%	30	62.50%

EPO: erythropoietin; LDH: lactate dehydrogenase.

**Table 3:** Univariate logistic regression analysis of various factors included in the study and risk of developing anemia at 21 days of life.

Characteristics *	OR (95% CI) *	Nagelkerke R Square	*p* Value	Anemia Incidence Rate **
Sex, male/female	0.490 (0.209–1.150)	0.036	0.101	78.13%/63.64%
GA	0.802 (0.668–0.963)	0.080	<0.05	
BW	0.999 (0.999–1.000)	0.057	<0.05	
APGAR score at 1 min	0.836 (0.680–1.026)	0.042	0.087	
APGAR score at 5 min	0.765 (0.566–1.034)	0.042	0.081	
Hb at 1 day of life	0.776 (0.635–0.949)	0.096	<0.05	
HCT at 1 day of life	0.930 (0.868–0.997)	0.061	<0.05	
RBC count at 1 day of life	0.316 (0.139–0.716)	0.116	<0.01	
aPTT at 1 day of life	0.993 (0.980–1.007)	0.012	0.356	
PT at 1 day of life	1.083 (0.970–1.208)	0.032	0.155	
LDH at 1 day of life	1.000 (0.999–1.001)	0.006	0.514	
Serum EPO at 1 day of life	1.093 (0.986–1.212)	0.045	0.090	
Iron administered during 7th–21st day of life, yes/no	2.400 (1.014–5.678)	0.053	<0.05	80.00%/62.50%
EPO administered in first 7 days of life, yes/no	0.440 (0.186–1.039)	0.047	0.061	63.27%/79.66%

GA, gestational age; BW, body weight; Hb, hemoglobin; HCT, hematocrit; RBC, red blood cell; aPTT, activated partial thromboplastin time; PT, prothrombin time; LDH, lactate dehydrogenase; EPO, erythropoietin; * ORs are displayed as crude values and include only the first subcategory in the case of sex, iron administration, and EPO administration, all dichotomous variables; ** incidence rate of anemia among neonates if their sex was male/female and if they underwent/did not undergo iron administration during the 7th to 21st day of life and EPO administration within the first 7 days of life.

**Table 4:** Univariate logistic regression analysis of various factors included in the study and risk of abnormal serum ferritin levels at 21 days of life.

Characteristics	OR (95% CI) *	Nagelkerke R Square	*p* Value	Abnormal Serum Ferritin Incidence Rate **
Sex, male/female	0.716 (0.309–1.659)	0.008	0.436	34.38%/27.27%
GA	0.910 (0.773–1.070)	0.017	0.254	
BW	0.999 (0.998–1.000)	0.024	0.173	
APGAR score at 1 min	0.952 (0.794–1.140)	0.004	0.590	
APGAR score at 5 min	0.924 (0.702–1.215)	0.004	0.570	
Hb at 1 day of life	0.975 (0.842–1.129)	0.002	0.732	
HCT at 1 day of life	0.960 (0.902–1.021)	0.023	0.192	
RBC count at 1 day of life	0.678 (0.347–1.328)	0.017	0.258	
aPTT at 1 day of life	1.002 (0.987–1.017)	0.001	0.783	
PT at 1 day of life	1.015 (0.931–1.106)	0.001	0.741	
LDH at 1 day of life	1.000 (0.999–1.001)	0.003	0.640	
Serum EPO at 1 day of life	0.981 (0.909–1.060)	0.003	0.632	
Iron administered during 7th–21st day of life, yes/no	0.690 (0.301–1.580)	0.010	0.380	35.00%/27.08%
EPO administered in first 7 days of life, yes/no	1.530 (0.668–3.504)	0.013	0.314	26.53%/35.59%

GA, gestational age; BW, body weight; Hb, hemoglobin; HCT, hematocrit; RBC, red blood cell; aPTT, activated partial thromboplastin time; PT, prothrombin time; LDH, lactate dehydrogenase; EPO, erythropoietin; * ORs are displayed as crude values and include only the first subcategory in the case of sex, iron administration, and EPO administration, all dichotomous variables; ** incidence rate of abnormal serum ferritin level among neonates if their sex was male/female and if they underwent/did not undergo iron administration during the 7th to 21st day of life and EPO administration within the first 7 days of life.

**Table 5:** Univariate logistic regression analysis of various factors included in the study and risk of abnormal serum iron levels at 21 days of life.

Characteristics	OR (95% CI) *	Nagelkerke R Square	*p* Value	Abnormal Serum Iron Incidence Rate **
Sex, male/female	1.013 (0.451–2.274)	0.000	0.976	65.63%/65.91%
GA	1.068 (0.913–1.250)	0.009	0.413	
BW	1.001 (1.000–1.002)	0.054	<0.05	
APGAR score at 1 min	0.905 (0.762–1.074)	0.007	0.253	
APGAR score at 5 min	0.887 (0.680–1.157)	0.010	0.377	
Hb at 1 day of life	1.010 (0.877–1.163)	0.000	0.891	
HCT at 1 day of life	1.036 (0.976–1.100)	0.018	0.248	
RBC count at 1 day of life	1.446 (0.752–2.783)	0.016	0.269	
aPTT at 1 day of life	1.009 (0.993–1.025)	0.020	0.256	
PT at 1 day of life	0.999 (0.921–1.084)	0.000	0.988	
LDH at 1 day of life	1.001 (1.000–1.002)	0.027	0.149	
Serum EPO at 1 day of life	1.018 (0.944–1.098)	0.003	0.646	
Iron administered during 7th–21st day of life, yes/no	0.772 (0.348–1.716)	0.005	0.526	68.33%/62.50%
EPO administered in first 7 days of life, yes/no	1.443 (0.649–3.205)	0.010	0.368	61.22%/69.49%

GA, gestational age; BW, body weight; Hb, hemoglobin; HCT, hematocrit; RBC, red blood cell; aPTT, activated partial thromboplastin time; PT, prothrombin time; LDH, lactate dehydrogenase; EPO, erythropoietin; * ORs are displayed as crude values and include only the first subcategory in the case of sex, iron administration, and EPO administration, all dichotomous variables; ** incidence rate of abnormal serum iron level among neonates if their sex was male/female and if they underwent/did not undergo iron administration during the 7th to 21st day of life and EPO administration within the first 7 days of life.

**Table 6:** Multivariate binomial logistic regression analysis of protective and risk factors previously associated with anemia in the study.

Characteristics	AOR (95% CI) *	*p* Value
GA	0.952 (0.706–1.283)	0.745
BW	0.999 (0.998–1.001)	0.401
Hb at 1 day of life	0.669 (0.295–1.515)	0.335
HCT at 1 day of life	1.170 (0.864–1.585)	0.310
RBC count at 1 day of life	0.351 (0.073–1.698)	0.193
Iron administration during 7th–21st day of life	2.749 (1.064–7.106)	<0.05

AOR, adjusted odds ratio; GA, gestational age; BW, body weight; Hb, hemoglobin; HCT, hematocrit; RBC, red blood cell; * ORs are displayed as adjusted values (AORs) and include only the first subcategory (yes) in the case of iron administration.

## Data Availability

The raw data are available upon request and have not been published to limit the availability of sensitive biometric personal information as per EU GDPR law.
